# Familiarity and Interest in Working with Livestock Decreases the Odds of Having Positive Attitudes towards Non-Human Animals and Their Welfare among Veterinary Students in Italy

**DOI:** 10.3390/ani8090150

**Published:** 2018-08-22

**Authors:** Chiara Mariti, Federica Pirrone, Mariangela Albertini, Angelo Gazzano, Silvana Diverio

**Affiliations:** 1 Dipartimento di Scienze Veterinarie, Università di Pisa, 56124 Pisa, Italy; angelo.gazzano@unipi.it; 2 Dipartimento di Medicina Veterinaria, Università degli Studi di Milano, 20133 Milano, Italy; federica.pirrone@unimi.it (F.P.); mariangela.albertini@unimi.it (M.A.); 3 Dipartimento di Medicina Veterinaria, Università degli Studi di Perugia, 06126 Perugia, Italy; silvana.diverio@unipg.it

**Keywords:** animal attitude scale, animal welfare, attitude towards animals, livestock, pets, veterinary students

## Abstract

**Simple Summary:**

Veterinary students are the future generation of veterinary practitioners. Therefore, their attitudes towards non-human animals and animal welfare have a substantial impact on animal welfare itself. This study investigated the attitudes of veterinary students in Italy. The findings suggest that factors such as female gender, familiarity with pets, or the intention to work with pet species, as well as an animal-free diet, and membership in an animal rights association are the predictors of a very positive attitude towards animals and their welfare. Familiarity and intention to work with livestock is instead associated with a less positive attitude. Students also considered the freedom to express normal behaviours and the freedom from fear and distress to be less important for livestock than for pets. Veterinary curricula should take these findings into account to improve the attitudes of students in order to improve animal welfare, especially the welfare of livestock.

**Abstract:**

We investigated the attitudes of veterinary students towards animals and their welfare in Italy. Regression analyses revealed predictors that are significant in differentiating students’ scoring tendency based on their gender, familiarity, and intention to work with a specific animal species, type of diet, and membership in an animal rights association. Female students, who were mostly familiar with pets and aspired to work with species other than livestock, following an animal-free diet and being a member of an animal rights association, had a significantly greater odds of having a high Animal Attitude Scale score (AAS), i.e., very positive attitude towards animals, versus a less positive attitude. Conversely, the familiarity with livestock and preference for working with livestock significantly increased the odds of a low AAS. Overall, students considered all of the Brambell Report’s Five Freedoms important for animal welfare protection. However, students scored higher for companion animals than for livestock, particularly regarding the freedom to express normal behaviour and the absence of fear and distress. This study suggests that veterinary students place less importance on the psychological aspects of welfare for livestock, and there is a tendency for students who are mostly familiar, or aspire to work, with livestock to have a less positive attitude towards non-human animals and their welfare. These findings should be considered within the veterinary educational curriculum due to their potential impact on animal welfare.

## 1. Introduction

Animal welfare has received increasing interest over the last decades, and it is now debated in the social, political, ethical, and scientific fields. Previously, there was great interest in the welfare of animals that are raised in intensive farm systems, which received strong criticism by the public [1] and led to relevant changes in European legislation [2] Then, the interest for animal welfare turned to other species and other issues that are related to the relationship of people with non-human animals, such as their use, moral consideration [3], mental capacities [4], and ability to feel pain [3].

Veterinary practitioners have been ranked among the professional figures that are best positioned to ensure animal welfare [5]. They are perceived as guarantors of animals’ health and thereby, their well-being [6], and they have been traditionally considered as a reference for public opinion and policy design. When considering their paramount role in ensuring and protecting animal welfare, some research over previous years has investigated different features of the attitudes of veterinary practitioners towards animals and their welfare, and the factors that may affect these attitudes [7,8,9,10]. Some studies have focused on veterinary students, who represent future veterinarians. Such studies, on the one hand, have confirmed the relevance of gender and previous experience with animals [11,12]. On the other hand, several studies have warned about the loss of empathy in veterinary medicine students and a possible detached attitude towards patients, especially in their senior years [11,13,14,15]. The latter is probably not related to a decline in students' beliefs in animal sentience [16], but similarly, to what occurs in human medicine students, to a sort of self-protection by becoming less empathetic with patients [17,18].

However, veterinarians are obliged to promote good animal welfare [19], which is one major step on the path to improving preventive veterinary care. The current study investigated whether the attitudes of future veterinarians in three Italian Universities were compatible with the promotion of good animal welfare. This was achieved by surveying veterinary students to identify the individual and experiential factors that may affect their attitudes towards non-human animals and their well-being.

## 2. Materials and Methods 

### 2.1. Collection of Data

The collection of data was performed using a questionnaire distributed by the authors in the three Italian veterinary schools where they are employed (Pisa, Milan, and Perugia). Paper questionnaires were distributed to all the students of each year course in the classroom at the beginning of the academic year 2016–2017 (1st week of lectures). Before completing the questionnaire, students were debriefed about the aim of the study, the anonymity, and the fact that the completion implied their consent to participate in the study. A few questionnaires were returned blank or mostly incomplete (*n* = 7).

The questionnaire (presented in Appendix A) was composed mainly of multiple choice items, divided into three sections. The first section (questions 1–9) investigated the demographic features of respondents, adapting items from the questionnaire that was used by Serpell [12] and kindly provided to us by the author: age, gender, year of course, origin, previous experience with animals, membership to an animal rights association, food restrictions, and preferred employment after graduation. The second section (questions 10–11) focused on the perception of the students about each of the Five Freedoms for the protection of livestock and pet animal welfare. The third section (question 12) consisted of items that aimed at assessing the students’ attitudes towards non-human animals. The latter items were taken from questionnaires in English previously used in scientific literature and translated into Italian following the procedure of back-translation. The questionnaire used for the current study also included the 20-item Animal Attitude Scale (AAS), a validated scale [20], as translated and modified by Gazzano [9].

### 2.2. Participants Demographics

We collected valid reports from 876 students (92.6% of questionnaires received, for a total of 946), quite evenly distributed between provenances: Pisa 39.4%, Milan 34.6%, and Perugia 26.0%. Respondents ranged in age from 18 to 45 years, with a mean age of 22 ± 0.10 years (mean ± standard error), and the majority were female (75.5%). There was a good spread across the course years, with the highest percentage of students attending the second year (31.2%). Eighteen per cent of respondents attended the first year, 19.3% were third-year students, 17.0% were fourth-year students, 13.1% were fifth-year students, and only 1.1% were attending a supplementary year of study. 

Participants came from all over Italy, although the majority came from the Central regions (46.4%) or from Northern Italy (41.1%), and only 10.0% had lived in the Southern regions. Just 2.5% of the sample came from a country other than Italy. As for the kind of environment, respondents had mostly lived in urban (42.4%), rather than rural (26.7%) or suburban (29.1%), settings. 

Most students had had ongoing experience in caring (keeping or owning) for dogs (23.4%), cats (18.6%), small mammals (13.9%), or fish (12.2%). About 9% of the students reported continuous experience with birds, 6.6% with poultry, and 6% with horses/ponies. A smaller proportion of respondents (10.0%) cared for other species (amphibians, pigs, ruminants, reptiles) on an ongoing basis. 

Almost one-third (32.5%) of students intended to work with dogs after graduation, and over one-third (34.2%) aspired to work in mixed practice (e.g., production animals and pets). About 13% of respondents intended to work in the area of horses and farm animals, 7.2% aspired to work in exotic practice, and 12.8% wished to be involved in other working areas once graduated.

While almost three-quarters of veterinary students (71.6%) were omnivorous, 10.9% did not eat meat, and 10% ate neither meat nor fish for ethical reasons. Only 2.8% of students were vegan and 4.5% followed other types of diet. The vast majority of respondents (88.6%) were not members of any animal rights association. 

### 2.3. Statistical Analysis

Statistical analyses were performed while using SPSS version 25.0 (IBM Corp., Armonk, NY, USA). Values are reported as means ± SE. The total scores were calculated and Cronbach’s alpha was used to estimate internal consistency [21]. For ease of interpretation, the scores were converted to the percentage of maximum possible (POMP) [22], where 0 and 100 represent the lowest and highest possible scale scores, respectively. 

Using non-parametric statistics (Mann-Whitney and Kruskal-Wallis with Bonferroni’s correction tests), we tested for differences in scoring the tendency of students as a preliminary screening. Ordinal Logistic Regression (OLR) models were then developed to assess the association between the AAS score (dependent variable, Y) and each of the potential risk factors (independent variables, X) for which there were significant differences in the answering tendency of students. Model Fitting was tested with the −2 log likelihood (−2LL) method for the intercept-only and final models. The Wald χ^2^ test was performed to evaluate which predictors were statistically significant. The odds ratio (OR) for the predictors was calculated to evaluate the strength of such relationships. The Test of Parallel Lines was used to assess the proportional odds assumption. A two-sided *p* < 0.05 was considered to be statistically significant.

## 3. Results

### 3.1. Scores on Brambell Report’s Five Freedoms 

Concerning livestock, the students’ average score on the Brambell Report’s Five Freedoms was 23.22 ± 0.09 out of 25, with a good Cronbach’s alpha score of 0.84. As for companion animals, the score was 23.47 ± 0.08 out of 25, showing very good reliability (Cronbach’s alpha = 0.86). A statistical difference was found between these two scores (Mann-Whitney U = 357813, *p* = 0.008) and the pairwise comparisons revealed, that, in particular, students consider that the freedom to express normal species-specific behaviour (mean score 4.60 ± 0.021 vs. 4.48 ± 0.023, companion animals vs. livestock, Mann-Whitney U = 350639, *p* = 0.001) and the freedom from fear and distress (mean score 4.72 ± 0.019 vs. 4.63 ± 0.021, companion animals vs. livestock, Mann-Whitney U = 352866, *p* = 0.001) are less important for the welfare of livestock than for companion animals. Female students had higher scores for both companion (23.70 ± 0.09, Mann-Whitney U = 57.05, *p* = 0.001) and livestock (23.50 ± 0.09, Mann-Whitney U = 53560, *p* = 0.001) than males (companion animals 22.73 ± 0.21 and livestock 22.32 ± 0.21). The gender was then submitted to OLR analyses. The likelihood ratio test revealed an improvement over the intercept-only model, demonstrating that the logistic model provided a better fit to the data both for production (−2 log likelihood of the intercept-only model = 1905.15; −2 log likelihood of the final model = 1836.50, χ^2^ = 68.64, df = 22, *p* = 0.001) and companion animals (−2 log likelihood of the intercept-only model = 1748.96; −2 log likelihood of the final model = 1693.78, χ^2^ = 55.171, df = 22, *p* = 0.001). 

The odds for the females scoring higher on production and companion animals were 2.24 and 2.61 greater than the odds for males, respectively (Table 1). The proportional odds assumption held because the significance of Chi-Square statistic in the Test for Parallel Lines was >0.05 (companion animals −2 log likelihood = 1401.13, χ^2^ = 292.65, df = 286, *p* = 0.38; livestock −2 log likelihood = 1538.18, χ^2^ = 298.32, df = 286, *p* = 0.29).

### 3.2. Animal Attitude Scale Scores 

The participants in this study showed a mean score of 66.88 ± 0.28 out of 100 on the AAS, with a good Cronbach’s alpha score of 0.81.

The scoring tendencies differed significantly by gender, year of course, familiarity with an animal species, favourite animal species to work with, diet, and membership of an animal rights organization. In particular, females scored higher than males (68.40 ± 0.28 vs. 62.17 ± 0.63, Mann-Whitney U = 41316, *p* = 0.001), as did students with continuous experience with dogs (67.34 ± 0.31, Mann-Whitney U = 50808, *p* = 0.001), cats (67.36 ± 0.34, Mann-Whitney U = 96323, *p* = 0.009), or fish (67.55 ± 0.38, Mann-Whitney U = 98373, *p* = 0.042) when compared to those without a particular experience with these species (65.10 ± 0.60, 66.06 ± 0.46, 66.42 ± 0.41 for dogs, cats, and fish, respectively). On the contrary, students who were familiar with ruminants had a lower score (64.50 ± 1.07) when compared to those who were not (67.11 ± 0.25) (Mann-Whitney U = 25223, *p* = 0.014).

At the same time, students who aspired to work with livestock after graduation had lower scores than students who intended to work with any other species (Kruskal-Wallis test χ^2^ = 54.646, df = 4, *p* = 0.001). 

Students who were members of an animal rights organization had significantly higher scores (75.29 ± 1.96) than those who were not (66.35 ± 0.29) (Mann-Whitney U = 8701, *p* = 0.001).

These variables, for which there were differences in the scoring tendencies of students, were submitted to the OLR analysis. The explanatory variables improved the model, because unexplained variation decreased from 3921.98 in the model with only the intercept, to 3634.60, and the difference (287.38) was statistically significant (*p* < 0.001).

There was strong evidence of an association between the AAS total score and the following variables: gender, familiarity with a species, being a member of an animal rights association, following a particular diet for ethical reasons, and the willingness to work with a specific species after graduation (Table 2). Females were over twice as likely to score high on the AAS than males. Students who were familiar with dogs and cats were approximately 1.5 times more likely to have a higher AAS score than students who were not familiar with these species, while familiarity with ruminants decreased the odds of scoring high on the AAS by 1.7 times. Being a member of an animal association increased the odds of having a higher level of AAS score by a factor of 4.4. 

The results regarding the predictor “diet” show that the students who did not eat some type of animal product for ethical reasons had a higher probability of obtaining high scores. The more animal products students that eliminated from their diet, the higher the probability of obtaining high scores. Lastly, on average, the willingness to work with horses, pets, exotic animals, or mixed species, once graduated, increased the odds of having a higher AAS score by approximately three times when compared to those willing to work with livestock.

The −2LL parallel regression assumption yielded χ^2^ statistics > 0.05, indicating that the proportional odds assumptions for the full-model was upheld (−2 log likelihood = 2176.01, χ^2^ = 1458.60, df = 1584, *p* = 0.989). 

## 4. Discussion

An increasing number of studies have documented that attitudes towards non-human animals, the perception of their welfare, and levels of empathy towards them can be influenced by a multitude of factors. These factors can be either features of the person or of the non-human animal.

Concerning how the features of a person affect his/her attitudes towards animals, many factors have been investigated and have been shown to be relevant. Among these, gender is of particular importance. The findings of the current study are in agreement with previous research, showing that female gender is commonly associated with more empathetic views towards animals, more concern for animal welfare issues, and, generally speaking, better attitudes towards animals when compared to male gender [20,23,24,25]. Although this gender bias is common, it can vary in young people [26] and cultural contexts [27,28,29]. Similarly, previous studies on veterinary students in the USA, Australia, and New Zealand have confirmed that female students have more positive attitudes towards animal welfare [12,30], whilst divergent results were found in Asian veterinary students [31,32].

Previous experience with animals is another factor that significantly influences people’s attitudes towards animals. As previously reported in veterinary students [12], as well as in other populations [20,24,33], we found a link between having experience in farm/rural context and a reduced concern for animal rights and animal welfare issues (e.g., less aversion to hunting, to the use of traps and live animals for medical training, and, in general, a more utilitarian view). Also, the willingness to work with livestock animals was associated with less positive attitudes towards non-human animals and their welfare. This finding is in line with a previous study that was performed in the USA [19], in which veterinary students aspiring to work with food animals considered more procedures to be humane for all species than did students aspiring to work with small animals. It is likely that these retrospective and prospective factors are related to one another, as the familiarity with a certain species increases the likelihood of working with it [5,12].

The most remarkable findings of the current study concern the features of the animals: the results suggest that veterinary students in Italy show different attitudes towards the welfare different species depending on the common use of the species. 

Specifically, respondents considered the freedom to express normal species-specific behaviours and the freedom from fear and distress less important for the welfare of livestock animals when compared to the welfare of pets. Previous research on different human populations has found that species that are phylogenetically close to man (apes) or that display human-like behaviours and habits (e.g., dogs and cats), as well as animals that are considered to be beauty symbols or rare and vulnerable, tend to evoke more empathetic responses, and therefore, are likely to be preferred and better treated when compared to more distant animals (reptiles, fishes, and invertebrates) [26,34,35,36,37,38]. The utility that certain animals have for man does not seem to evoke this kind of response—lab mice and rats, despite their utility and relative closeness, do not evoke affection or concern for their condition [39]. It is remarkable that in the current study, a different attitude towards livestock versus pet welfare was found for the Brambell Report’s Five Freedoms with regard to those freedoms that involve the psychological aspects of welfare [9]. As for other categories of people, even veterinary students seem to distinguish between “animals good to think with” and “animals good to eat” [40]. In fact, it is well known that loving animals and eating meat is a moral conflict (the meat-paradox [3]). However, this morally troublesome behaviour is somewhat resolved by categorizing certain animals as food, and thus seeing them as insensitive to pain and unworthy of moral consideration [41]. Something similar seems to also occur among veterinary students. For instance, veterinary students in the USA were more likely to believe that dogs and cats have cognitive abilities than farm animals, and they considered various procedures to be more humane for farm mammals than for dogs and cats [19]. The attitudes of veterinary students towards the welfare of different species of farm animals should also be found [5], and further research is required to better deal with these attitudes.

The species of an animal is also relevant for some of the risk factors found for the AAS score. In the current study, as already suggested by Serpell [12], previous experience with animals does affect the attitudes of veterinary students towards them, but the kind of influence varies according to the species. Here, we found that familiarity with companion animals—in terms of ownership—was associated with a higher level of conferral of emotions to animals [42], which appeared to lead to a more pro-animal attitude [43]. Menchetti [44] also suggested that different attitudes to owned animals could be influenced by the perception of their personalities by owners.

Overall, this study found great similarities to other studies that were carried out on the attitudes towards animals of veterinary students in other Western countries [11,12,19,30]. Previous studies have highlighted gaps in the teaching of animal welfare, which possibly impair veterinary competency in the area [45]. European Union (EU) legislation requires that veterinary surgeons acquire an adequate knowledge of the behaviour and protection of animals [46], highlighting the relevance of veterinary training for the prevention of some poor animal welfare issues [47]. This study suggests that future veterinarians who will work with livestock have a worse attitude towards their patients and a lower consideration for the psychological aspects of their patients’ welfare, when compared to future small animal veterinary surgeons. These points should be taken into account in the veterinary curricula, including in the education of veterinary students, where more effort to improve the attitudes towards non-human animals and animal welfare should be made. Veterinary students should be trained about issues relating to the physiological and ethological needs of farm animals, as well as to carry out an assessment of relevant welfare requirements and outcomes, and to promote interest among farmers in their welfare-related management and to support those with poor outcomes with remedial action [48]. Training veterinary students for these skills has to be carried out properly and effectively in order to improve the knowledge and attitude of future generations of veterinarians. This will indirectly increase farm animals’ welfare, preventing animal health-related threats and minimizing environmental impacts, in support of the EU Sustainable Development Strategy [49]. A more structured teaching animal welfare in veterinary curricula is also required for those students who will deal with small animals in the future [50], especially if their specialization does not include behaviour [9,50].

## 5. Conclusions

The findings of this study confirm that attitudes of veterinary students towards non-human animals and animal welfare in Italy, as in previously investigated countries, is largely influenced by the features of the respondent and of the animal. Familiarity with livestock animals as well as the willingness to work with them are risk factors for developing a less positive attitude. Previous studies highlighted a reduced sensitivity of veterinary students to non-human animals and their welfare over the years of their course [11,13,14]. Hazel [15] found that attendance to a course on animal welfare was effective in improving attitudes towards certain animal species, but these positive results are not universally shared [5,51]. Taken together, all of this knowledge suggests that more effort should be devoted in veterinary curricula to indirectly enhance the welfare of farm species to convince students that, in today's livestock farming, it is not just about the survival of the animals, but, above all, it is about the quality of their lives [52].

## Figures and Tables

**Table 1:** Parameter estimates according to the scores on the Brambell Report’s Five Freedoms.

Score	Predictor	B	SE	Wald	df	Sig.	OR	95% CI for OR	
								Lower Limit	Upper Limit
Livestock	sex = female	0.81	0.19	17.86	1	0.001	2.24	1.54	3.25
	sex = male	0 ^a^					1		
Companion animals	sex = female	0.96	0.186	26.47	1	0.001	2.61	1.81	3.77
	sex = male	0 ^a^					1		

Significance: *p* < 0.05. B: regression coefficient, SE: standard error of the mean, OR: odds ratio, CI: confidence interval. ^a^ This parameter was set to zero because it is redundant.

**Table 2:** Ordinal Logistic Regression model result predicting students Animal Attitude Scale (AAS) scoring tendencies.

Predictor	B	SE	Wald	df	Sig.	OR	95% CI for OR	
							Lower Limit	Upper Limit
Sex = female	1.006	0.169	35.444	1	0.001	2.736	1.964	3.810
Sex = male	0 ^a^					1		
Familiarity with dogs = yes	0.495	0.176	7.872	1	0.005	1.640	1.161	2.320
Familiarity with dogs = no	0 ^a^					1		
Familiarity with cats = yes	0.391	0.143	7.526	1	0.006	1.479	1.118	1.960
Familiarity with cats = no	0 ^a^					1		
Familiarity with ruminants = no	0.533	0.248	4.608	1	0.032	1.703	1.047	2.770
Familiarity with ruminants = yes	0 ^a^					1		
Animal rights association membership = yes	1.476	0.505	8.526	1	0.004	4.375	1.625	11.782
Animal rights association membership = no	0 ^a^					1		
Vegan diet	3.053	0.528	33.397	1	0.001	21.172	7.518	59.623
Meat and fish-free diet	1.768	0.541	53.983	1	0.001	5.862	3.657	9.395
Meat-free diet	1.422	0.231	37.826	1	0.001	4.144	2.634	6.520
Omnivorous diet	0 ^a^					1		
Career preference = horses	1.730	0.370	21.903	1	0.001	5.643	2.734	11.658
Career preference = pets	1.668	0.312	28.497	1	0.001	5.3011	2.873	9.789
Career preference = mixed species	1.395	0.310	20.259	1	0.001	4.033	2.197	7.402
Career preference = exotic species	1.389	0.379	13.450	1	0.001	4.012	1.910	8.431
Career preference = others	0.533	0.248	4.608	1	0.032	1.703	1.047	2.770
Career preference = livestock	0 ^a^					1		

Significance: *p* < 0.05. B: regression coefficient. SE: standard error of the mean, OR: odds ratio, CI: confidence interval. ^a^ This parameter was set to zero because it is redundant.

## Appendix A

1. Which year of course are you attending: ☐ 1 ☐ 2 ☐ 3 ☐ 4 ☐ 5 ☐ Other______

2. Age: ______

3. Gender: ☐ Male ☐ Female

4. Which of the following would best describe your background?

☐ Predominantly rural ☐ Predominantly urban ☐ Predominantly suburban ☐ Other______

5. Where did you mainly live before attending the University?

☐ Italian Region ________ ☐ Foreign Country_____

6. With which of the following kinds of animals have you had continued experience (please check all that apply)?

☐ Dog(s) ☐ Cat(s) ☐ Horse(s), Pon(ies) ☐ Cattle, sheep, goat(s) ☐ Pig(s) ☐ Poultry ☐ Small mammals (rabbits, hamsters, etc.) ☐ Cage birds/parrots ☐ Reptiles/amphibians ☐ Other______

7. From the following list, please indicate your preferred type of employment after you graduate from veterinary school: ☐ Farm animals (cattle, pigs, poultry, etc.) ☐ Equine practice ☐ Small animal practice ☐ Mixed practice ☐ Exotics/special species ☐ Other __________

8. Have you ever been on a diet excluding some food of animal origin for ethical reasons (not for health reasons or taste preferences): ☐ No ☐ Yes, a diet without any product of animal origin ☐ Yes, a diet without meat and fish ☐ Yes, a diet without meat ☐ Yes, other ___________

9. Have you ever been a member of an animal rights association? ☐ No ☐ Yes, which?___________

10. In your opinion, how important for the welfare of farm animals are each of the following freedoms listed in the Brambell report in 1965?

	**Not at All Important**	**Slightly Important**	**Fairly Important**	**Very Important**	**Extremely Important**
Freedom from thirst, hunger, and poor nutrition					
Freedom from discomfort (suitable environment)					
Freedom from pain, injury, or disease					
Freedom to express (most) normal behaviour					
Freedom from fear and distress					

11. In your opinion, how important for the welfare of pet animals are each of the following freedoms listed in the Brambell report in 1965?

	**Not at All Important**	**Slightly Important**	**Fairly Important**	**Very Important**	**Extremely Important**
Freedom from thirst, hunger, and poor nutrition					
Freedom from discomfort (suitable environment)					
Freedom from pain, injury, or disease					
Freedom to express (most) normal behaviour					
Freedom from fear and distress					

12. Please indicate your level of agreement/disagreement with each of the following statements

	**Strongly Agree**	**Agree**	**Undecided**	**Disagree**	**Strongly Disagree**
It is morally wrong to hunt wild animals just for sport					
I do not think that there is anything wrong with using animals in medical research					
There should be extremely stiff penalties, including jail sentences for people who participate in dog-fighting					
Wild animals, such as mink and raccoons, should not be trapped and their skins made into fur coats					
There is nothing morally wrong with hunting wild animals for food					
I think people who object to raising animals for meat are too sentimental					
Much of the scientific research done with animals is unnecessary and cruel					
I think it is perfectly acceptable for cattle and hogs to be raised for human consumption					
Basically, humans have the right to use animals as we see fit					
The slaughter of whales and dolphins should be immediately stopped immediately even if it means some people will be put out of work					
I sometimes get upset when I see wild animals in cages in zoos					
In general, I think that human economic gain is more important than setting aside more land for wildlife					
Too much fuss is made over the welfare of animals these days when there are many human problems that need to be solved					
Breeding animals for their skins is a legitimate use of animals					
Some aspects of biology can only be learned through dissecting preserved animals such as frogs					
Continued research with animals will be necessary if we are to ever conquer diseases such as cancer, heart disease and AIDS					
It is unethical to breed purebred dogs for pets when millions of dogs are killed in animal shelters each year					
The production of inexpensive meat, eggs, and dairy products justifies maintaining animals under crowded conditions					
The use of animals such as rabbits for testing the safety of cosmetics and household products is unnecessary and should be stopped					
The use of animals in rodeos and circuses is cruel					
